# Impacts of environmental pressures on the reproductive physiology of subpopulations of black rhinoceros (*Diceros bicornis bicornis*) in Addo Elephant National Park, South Africa

**DOI:** 10.1093/conphys/cot034

**Published:** 2014-02-03

**Authors:** Elizabeth W. Freeman, Jordana M. Meyer, Jed Bird, John Adendorff, Bruce A. Schulte, Rachel M. Santymire

**Affiliations:** 1 New Century College, George Mason University, Fairfax, VA, USA; 2 Department of Conservation and Science, Lincoln Park Zoo, Chicago, IL, USA; 3 South African National Parks, Addo Elephant National Park, Addo, South Africa; 4 Department of Biology, Western Kentucky University, Bowling Green, KY, USA

**Keywords:** Androgens, *Diceros bicornis bicornis*, inter-calving interval, progestagens

## Abstract

Black rhinoceros are an icon for international conservation, yet little is known about their physiology due to their secretive nature. To overcome these challenges, non-invasive methods were used to monitor rhinoceros in two sections of Addo Elephant National Park, South Africa, namely Addo and Nyathi. These sections were separated by a public road, and the numbers of elephants, predators and tourists were higher in Addo. Faecal samples (*n* = 231) were collected (from July 2007 to November 2010) from known individuals and analysed for progestagen and androgen metabolite (FPM and FAM, respectively) concentrations. As biotic factors could impact reproduction, we predicted that demographics, FPM and FAM would vary between sections and with respect to season (calendar and wet/dry), climate and age of the rhinoceros. Mean FPM concentrations from pregnant females were seven times higher (*P* < 0.05) than samples from non-pregnant rhinoceros. Positive relationships were found between monthly temperatures and FPM from non-pregnant females (*r*^2^ = 0.25, *P* = 0.03) and the percentage of calves born (*r* = 0.609, *P* = 0.04). Although FAM peaked in the spring, when the majority of calves (40%) were conceived, no seasonal patterns in male androgen concentrations were found with respect to month of conception and parturition. Females in Addo had a longer inter-calving interval and were less likely to be pregnant (*P* < 0.05) compared with those in Nyathi. The biotic stressors (e.g. predators and more competitors) within Addo section could be affecting the reproductive physiology of the rhinoceros negatively. Enhanced knowledge about how black rhinoceros populations respond to environmental stressors could guide management strategies for improving reproduction.

## Abstract (precis)

We conducted the first physiological study of a free-ranging population of the black rhinoceros subspecies (Diceros bicornis bicornis). We discovered that environmental factors impact reproductive physiology (e.g. gonadal hormones, inter-calving intervals) of rhinos. Further knowledge about what affects reproduction in rhinos could enhance propagation to counterbalance high rates of poaching.

## Introduction

The black rhinoceros (‘rhino’; *Diceros bicornis*) is an elusive creature that captivates many people as a charismatic reminder of prehistoric times and, in recent years, as an iconic species for conservation efforts. Primarily due to poaching for their horns, the black rhino population suffered one of the most dramatic declines of the 20th century (Garnier *et al.*, 2001), decreasing by 97.6% from 1960 to 1995 to a critically low size of 2410 individuals (Emslie, 2012). Since 1995, black rhinos have been listed as critically endangered by the IUCN Red List even though populations continent-wide have been steadily increasing to an estimated size of ∼4880 individuals by the end of 2010 (Emslie, 2012). South Africa's population of black rhinos was reduced to 110 animals in 1935 (0.1% of the continental population), yet increased to 1750 animals by 2008, ∼35% of the African population, due to successful conservation management efforts coinciding with population declines in other range countries (Knight *et al.*, 2011).

Aspects of black rhino physiology and behaviour that affect reproductive success remain poorly understood due to their elusive nature. For instance, their long gestation, solitary social structure and typically low densities in thick habitat make monitoring reproduction difficult (Garnier *et al.*, 2002). The use of non-invasive techniques to collect faecal samples can facilitate the monitoring of reproduction in free-ranging populations without disturbing or even seeing individuals (Garnier *et al.*, 2002; Monfort, 2003) and can provide information about the onset of sexual maturity, ovulation, pregnancy, parturition and fetal/neonatal loss (Berkeley *et al.*, 1997; Garnier *et al.*, 2002). Demographic studies of free-ranging populations (e.g. sexual maturity and calving intervals) and monitoring the physical condition of individuals through non-invasive hormone analyses can provide key information on how populations are responding to the environment and what management changes are necessary to improve reproductive success (Hitchins and Anderson, 1983).

Only a few non-invasive endocrine studies have been conducted on wild black rhino, including a field pregnancy test (MacDonald *et al.*, 2008) and an evaluation of pregnancy and oestrous hormones, as well as the influence of season on reproduction (Garnier *et al.*, 1998, 2002). Prolonged periods with wild-caught rhinoceros held in bomas prior to translocation caused a decline in faecal androgen (FAM) and progestagen metabolite (FPM) concentrations, indicating a biological cost associated with management (Linklater *et al.*, 2010). Additionally, faecal hormone concentrations and scent-marks were evaluated relative to distance travelled after those rhinos were translocated (Linklater *et al.*, 2006). Changes in faecal gonadal hormone metabolites with respect to translocations suggest that management decisions can negatively impact reproduction in black rhinoceros.

Although only a few endocrine studies have focused on free-ranging rhinos, additional research has been carried out in zoos (Schwarzenberger *et al.*, 1993, 1996; Berkeley *et al.*, 1997; Wasser *et al.*, 2000; Brown *et al.*, 2001; Radcliffe *et al.*, 2001; Carlstead and Brown, 2005). Schwarzenberger *et al.* (1996) found no differences in faecal progestagen metabolite concentrations between zoo-managed *D. b. michaeli* and *D. b. minor*. Given that zoo animals are managed with different environmental and dietary conditions from those of wild black rhinos, we cannot assume that hormonal patterns are similar between wild and captive populations. No data have been published on faecal endocrine concentrations in *D. b. bicornis* from either free-ranging or zoo-based populations.

Female black rhinos most frequently have oestrous cycles characterized by periods of elevated FPM concentrations (luteal phase) for 14–19 days and periods of low concentrations (follicular phase; 8–14 days) for a total duration of ≤40 days (type I cycle; Garnier *et al.*, 2002). Type II cycles extend >40 days due to extended luteal (19–36 days) and follicular phases (11–36 days) and can be either anovulatory or the result of a lost embryo (Garnier *et al.*, 2002). Progestagen concentrations during the luteal phase are 1.3–2 times higher than in the follicular phase (Schwarzenberger *et al.*, 1993; MacDonald *et al.*, 2008). Hormone measurements obtained from faeces can be used to diagnose pregnancy in black rhinos after about 3 months, when FPM concentrations rise 4–10 times higher than non-pregnant values and remain elevated until parturition (Schwarzenberger *et al.*, 1993, 1996; Berkeley *et al.*, 1997; Garnier *et al.*, 1998; MacDonald *et al.*, 2008).

Birth rates are challenging to monitor in free-ranging black rhino because of the difficulty in detecting newly born calves in natural conditions (Garnier *et al.*, 1998). However, it is known that free-ranging black rhino are polygynous (Garnier *et al.*, 2001; Christensen *et al.*, 2009); females take about 7 years to reach sexual maturity, have a gestation of 15 months (Hall-Martin and Penzhorn, 1977; Garnier *et al.*, 1998) and calving interval ranging from 2 to 5 years (Hall-Martin and Penzhorn, 1977; Hitchins and Anderson, 1983; Garnier *et al.*, 1998).

Less is known about male reproduction. Serum testosterone concentrations in zoo-based animals rise until males reach 8 years of age and then remain steady through advanced age (Christensen *et al.*, 2006). Males can sire young as early as 4.5 years of age in zoos and 7 years of age in the wild, and prime breeding years vary with respect to population demographics (Garnier *et al.*, 2001). Furthermore, zoo-based demographics affect male endocrinology; those housed with conspecifics (both males and females) have higher circulating testosterone concentrations than solitary males (Christensen *et al.*, 2009). Faecal androgen metabolite (FAM) concentrations have not been studied in free-ranging male black rhinos.

The present reproductive study took place in two sections of Addo Elephant National Park (AENP) in the Eastern Cape of South Africa, where the Southwestern arid subspecies of black rhino (*D. bicornis bicornis*) are managed. These sections vary with respect to inter-specific competition with elephants, predation pressures and levels of tourism, making AENP an ideal location to study the impact of ecology and management on the reproductive physiology of the black rhino. Black rhinos select their habitat based on a variety of factors, including the distance to water, the presence of roads and fences and the quality of available browse (Morgan *et al.*, 2009). Additionally, population models predict that competition with other browsers (e.g. elephants) and low precipitation rates could negatively impact reproductive success of black rhinos (Birkett, 2002). Reductions in availability of food, water and shelter, along with increased human disturbance, results in larger home ranges and lower black rhino reproductive success (Reid *et al.*, 2007). Thus, differences in competition for food, water and shelter between the AENP sections could affect hormonal activity and ultimately lead to differences in reproductive success among individuals. Expanding our knowledge about the reproductive physiology of free-ranging rhinos in the presence of these environmental stressors would enhance our ability to evaluate and improve the efficacy of conservation and management practices (Cooke *et al.*, 2013).

The purposes of this study were to use non-invasive techniques to monitor reproductive physiology of the black rhinos in the two sections of AENP, Addo and Nyathi. We investigated the following hypotheses: (i) high densities of elephants, predators and tourists in Addo section negatively impact black rhino reproduction in comparison to Nyathi; and (ii) reproductive success and hormonal activity vary with respect to season, temperature and precipitation. Investigation of the factors that negatively affect rhino reproduction will provide South African National Park (SANParks) officials with data about management changes that could be made to improve reproductive success. It is critically important to ensure that all mature black rhinos reproduce successfully, as poaching rates continue to rise. Furthermore, endocrine data on free-ranging *D. b. bicornis* will contribute to the growing field of conservation physiology (Cooke *et al.*, 2013).

## Methods

### Study area

Addo Elephant National Park (33°31′S, 25°45′E) is located in the Eastern Cape Province of South Africa, 60 km north-east of Port Elizabeth (Landman *et al.*, 2008). In 1931, it was proclaimed in order to protect the remaining elephants (*n* = 11) in the area (Penzhorn *et al.*, 1974; Hall-Martin and Penzhorn, 1977). The last endemic black rhinoceros (subspecies *D. b. bicornis*) in the Eastern Cape region was shot in 1858 (Hall-Martin and Penzhorn, 1977). Black rhino were first reintroduced to AENP in 1961, but were of the East African subspecies *D. b. michaeli* (Hall-Martin and Penzhorn, 1977). Starting in 1995, *D. b. michaeli* rhino were slowly removed and the indigenous subspecies *D. b. bicornis* were reintroduced to AENP.

The AENP consists of multiple sections, three of which contain black rhino. Our study focused on two ecologically similar sections, Addo (the Main Camp) and Nyathi, that consisted of endemic rich succulent thicket biome (Mucina and Rutherford, 2006; Landman *et al.*, 2008), including large tracts of dense vegetation interspersed with small, open areas (Hayward and Hayward, 2006).

The Addo section of AENP was expanded in late 2010, but for the majority of this study consisted of 11 500 ha of habitat ranging from open, grassy plains to xeric subtropical succulent thicket (Whitehouse and Hall-Martin, 2000). Addo receives on average 445 mm of rain per annum (SANParks, 2009). Natural water pans are filled by the seasonal rainfall, but water is also continuously supplied to seven man-made waterholes. Compared with Nyathi, the Addo area has a higher density of elephants, 3.58 elephants/km^2^ (J. M. Meyer, personal observation), has lion (*Panthera leo*) and spotted hyena (*Crocuta crocuta*; Hayward *et al.*, 2007), and also receives a greater number of tourists, 140 000 annually (Hayward and Hayward, 2011).

Nyathi (14 000 ha) is located a kilometre north of the Addo section, and the two are separated by fences and a public road. Nyathi is composed of grassland and thicket that receives an annual rainfall of 445–600 mm (SANParks, 2009). An ephemeral river, The Coerney, which flows for a few months after heavy rains, provides the main water source; additionally, multiple dams and pans are dispersed throughout Nyathi. The density of elephants in Nyathi is lower (0.71 elephants/km^2^; J. M. Meyer, personal observation) than Addo. Like Addo, Nyathi is rich in ungulates, but there are no large predators (i.e. lion or hyena). Additionally, Nyathi is not open to the general public; only visitors of two concessionaries (Riverbend Lodge and Nguni River Lodge) are allowed to traverse this section of the park. We do not have annual numbers of tourists for Nyathi, but these concessionaries combined hold ∼100 people per night, so even if full every night of the year, tourist numbers would have been a quarter (26%) of those in Addo.

### Rhinoceros population

**Figure 1.**
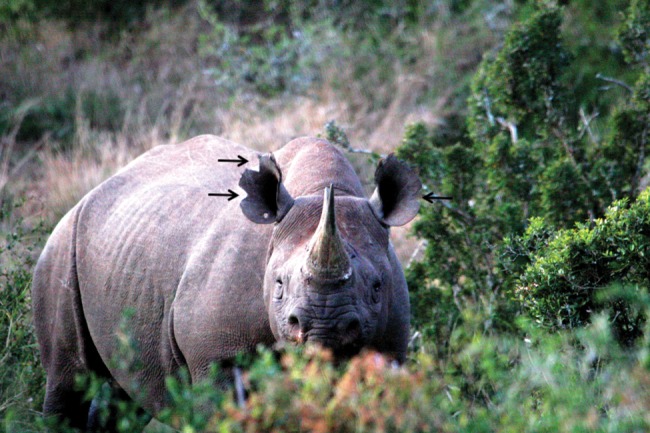
Photograph of an adult male black rhinoceros in the Nyathi section of Addo Elephant National Park, South Africa. Individuals can be identified by distinct anatomical features and a specific pattern of ear notches (black arrows). Photograph by J. Bird.

About 70% of South Africa's population of *D. b. bicornis* resides in AENP. At of the end of this study, the two focal sections of AENP contained 46 rhinos (Addo, *n* = 20, 0.17 rhino/km^2^; and Nyathi, *n* = 26, 0.18 rhino/km^2^) with similar population and age structures (Addo adults, five male (♂) and five female (♀); Addo subadults, one ♂ and three ♀; Addo calves, three ♂, one ♀ and one unsexed; Nyathi adults, seven ♂ and seven ♀; Nyathi subadults, six ♂ and two ♀; and Nyathi calves, one ♂, two ♀ and one unsexed). To assist in identifying the rhinos, each individual was darted and chemically restrained at ∼2–4 years of age, and given a name and a specific pattern of ear notches (Fig. 1). Individuals can be positively identified by other anatomical features, such as their horn, size and bodily scars prior to notching.

### Camera traps and sample collection

Camera traps (ScoutGaurd 550V and ScoutGaurd SG550, HCO, Norcross, GA, USA; and Wildview STC-TGL3IR, Grand Prairie, TX, USA) were erected in AENP at active middens and based on rhino sightings (Freeman *et al.*, 2014). When the camera was activated through a passive infrared motion detector, a high-quality digital photograph was quietly taken. A faecal sample was collected into a 12.7 cm ×17.8 cm, zip-top polyethylene bag (#EF28278D, Daigger, Vernon Hills, IL, USA) each morning that fresh faeces were found in the vicinity of a camera (Freeman *et al.*, 2014). When the surface of the faeces was still wet and no insect contamination had yet occurred, faecal samples were considered to be fresh (Garnier *et al.*, 1998, 2002). The identity of the individual that defaecated was determined by reviewing the images stored on the camera (Freeman *et al.*, 2014). Faeces were immediately stored at 4°C in a portable vehicle refrigerator until they were transported to our field laboratory for hormone extraction.

A total of 422 faecal samples were collected from black rhino in Nyathi (*n* = 339) and Addo (*n* = 83) between July 2007 and November 2010; however, only 231 faecal samples could be positively identified through visual identification or camera trap photograph. Although endocrine analyses were conducted on all of the samples, only results from known individuals (Nyathi, *n* = 184, 122 ♂ and 62 ♀; and Addo, *n* = 47, 22 ♂ and 25 ♀) are reported herein (Table 1).

**Table 1.** Black rhinoceros within Addo Elephant National Park from which faecal samples were non-invasively collected between 2007 and 2010

Rhino	Sex	Section	Age range (years; min–max)	Faecal samples (*n*)
ANK	Female	Addo	12	1^a^
CHO	Male	Addo	6	1
IFE	Male	Addo	6–7	7
KOR	Female	Addo	13	1
MOS	Female	Addo	4	1
MSC	Male	Addo	1	3
MSI	Female	Addo	7	7^a^
NAB	Female	Addo	7	5
NGA	Female	Addo	15–18	3^a^
NGC	Female	Addo	1	1
ONG	Male	Addo	12–14	4
PET	Male	Addo	12–13	6
TSW	Male	Addo	15–16	2
ZIM	Female	Addo	3	1
CAC	Male	Nyathi	0.5–2	6
CAT	Female	Nyathi	9–11	12^a^
DUL	Female	Nyathi	14–15	3^a^
HM	Male	Nyathi	8–9	3
INT	Female	Nyathi	1–2	2
JEC	Female	Nyathi	0.5	1
KAR	Male	Nyathi	27	4
KEN	Female	Nyathi	16–19	20^a^
LIM	Female	Nyathi	7–8	3
MAH	Male	Nyathi	4–5	2
MAL	Male	Nyathi	13–16	21
MAN	Male	Nyathi	2–4	11
MAZ	Female	Nyathi	1–4	16
MUN	Male	Nyathi	7–10	28
OMU	Male	Nyathi	7–8	3
RV	Male	Nyathi	16–18	20
THD	Male	Nyathi	3	2
THZ	Male	Nyathi	3–5	12
TSA	Female	Nyathi	18	1
TSC	Female	Nyathi	1	1
UBU	Female	Nyathi	5–6	3
VUK	Male	Nyathi	5	1

^a^Pregnant females.

### Faecal hormone monitoring

A field technique (Santymire and Armstrong, 2010) was used to extract hormones from the faeces. After thoroughly mixing the thawed samples, 0.5 ± 0.02 g of wet faeces was weighed on a Mettler-Toledo battery-powered balance (#PL202-S/00; accurate to 0.01 g) into a 16 mm ×100 mm polypropylene tube (#2332 and 2305, Perfector Scientific, Atascadero, CA, USA). A faecal slurry was created by homogenizing each sample with 5 ml of 70% propanol for 1 min using a microhomogenizer (Omni International, Marietta, GA, USA) with a clean, disposable hard tissue generator. The slurry was immediately poured through a ‘funnel-shaped’ filter paper (Whatman Grade 3; 9 cm diameter, cut in half), and a 1 ml aliquot of the filtered extract was transferred to a new 16 mm ×100 mm polypropylene tube. After the extract air dried, it was capped until reconstituted for analysis. Extract tubes were heated to 72°C for 30 min (under USDA permit #107647) and were shipped to the Davee Center for Endocrinology and Epidemiology at Lincoln Park Zoo (Chicago, IL, USA) for analysis of gonadal (progestagen and androgen) metabolite concentrations (Freeman *et al.*, 2014). Faecal extracts were reconstituted in 1 ml of dilution buffer (0.2 m NaH_2_PO_4_, 0.2 m Na_2_HPO_4_ and 0.14 m NaCl) with glass beads, vortexed briefly, sonicated for 20 min and further diluted for the enzyme immunoassays.

The FPM was analysed using an enzyme immunoassay of progesterone polyclonal antiserum (CL425; provided by C. Munro, Davis, CA, USA; 1:10 000 dilution) and horseradish peroxidase (1:40 000; Freeman *et al.*, 2014). Cross-reactivities of the progesterone antibody were previously reported (Graham *et al.*, 2001; Loeding *et al.*, 2011). The progesterone enzyme immunoassay was validated by demonstrating the following characteristics: (i) parallelism between binding inhibition curves of faecal extract dilutions (neat, 1:256) and the progesterone standard (*r* = 0.97); and (ii) significant recovery (>90%) of exogenous progesterone added to faecal extracts (1:1000; *y* = 1.04*x* − 2.10; *r*^2^ = 0.999). Assay sensitivity was 0.78 pg per well, and intra- and inter-assay coefficients of variation were <15%.

Faecal androgen metabolites were measured using a testosterone enzyme immunoassay (R156/7; also provided by C. Munro, Davis, CA, USA); the horseradish peroxidase and polyclonal antiserum were used at dilutions of 1:30 000 and 1:10 000, respectively. Antiserum cross-reactivities for testosterone were previously published (Santymire and Armstrong, 2010). The testosterone enzyme immunoassay was validated for the black rhino by demonstrating the following characteristics: (i) parallelism between binding inhibition curves of faecal extract dilutions (1:80–1:5120) and the testosterone standard (*r* = 0.993); and (ii) significant recovery (>90%) of exogenous testosterone (2.3–600 pg per well) added to faecal extracts (1:5000; *y* = 0.71*x* + 0.88; *r*^2^ = 0.999). Assay sensitivity was 2.3 pg per well, and intra- and inter-assay coefficients of variation were <15%.

### Data analysis

Black rhino were categorized into three age classes, namely calf (0–3 years), subadult (3–6 years) and adult (≥7 years of age). Calves are individuals that are still with their mothers and suckling until 3 years of age; after this time, they cease nursing and become independent. At ∼7 years of age, rhinos become reproductively active and are considered adults; all animals between weaning and reproductive maturity are considered subadults. Total monthly precipitation (in millimetres) and average monthly temperature data (in degrees Celsius; 2005–2012) were provided by the South African Weather Service (Walmer, South Africa). Rainfall within AENP does occur throughout the year, but there are peaks in February–March and October–November. The AENP wet season was thus defined as October–March and the dry season April–September.

Comparisons of age at first parturition (AFP) and inter-calving interval (ICI) between the two sections, Addo and Nyathi, were analysed using a Student's unpaired *t* test. When the month and year of birth of both the mother and calf were known, AFP was calculated to the month; otherwise, it was estimated to the nearest year. Inter-calving interval was determined as the interval (in months) between sightings of the mother with successive neonatal calves (Hitchins and Anderson, 1983).

Differences in total precipitation and average temperature among the months were assessed with analysis of variance (ANOVA). To determine whether distinct patterns existed for month of conception and parturition, the months of the year were converted into an angular direction on a circular scale. The uniform distribution of conception and parturition months around the circle was tested using the Rayleigh test (Zar, 1999; Di Betetti and Janson, 2001). Pearson correlation was used to investigate the relationships between climate (temperature and precipitation) and parturition and conception months. Linear regression was employed to measure the relationships between FPM and FAM and climate (temperature and precipitation).

The variables that predicted whether or not a female black rhino was pregnant were analysed with a binomial generalized linear model (GLM). Pregnant (yes/no) was examined with respect to the following factors: year, season, wet/dry, temperature, precipitation, age category and section. Linear mixed-effects (LME) models were used to determine which variables predicted the concentrations of FPM for females and FAM for males. Mixed-effects models can control for sources of between-individual heterogeneity, thus allowing for more accurate measurement of within-individual patterns in longitudinally measured data (Nussey *et al.*, 2008). Given that the model data included repeated measures from individual rhinos, animal identity was incorporated as a random effect. Female FPM concentrations were examined with respect to the following factors: year, calendar season (spring, summer, autumn and winter), wet/dry season, average monthly temperature, total monthly precipitation, age category, section (Nyathi and Addo) and pregnant (yes/no). Male FAM concentrations were examined with respect to all of the same variables except pregnant. Step-wise elimination of non-significant variables in the GLM and LME models was conducted, and reduced models were compared with the full model using smaller Akaike's information criteria (AIC) values as a guide for model selection.

Data were analysed with a *t* test, ANOVA or Pearson correlation using SigmaPlot (version 11.0, 2008; SPSS, Inc., Chicago, IL, USA). A Kolmogorov–Smirnov test was used for normality assumption testing and the Levene median test for equal variance assumption testing. For non-normally distributed data, a non-parametric test was used. The binomial GLM and LME model were analysed in the free statistical package R (R Development Core Team, 2012). For all analyses, *P* < 0.05 was considered significant, and data are reported as means ± SEM.

## Results

**Figure 2.**
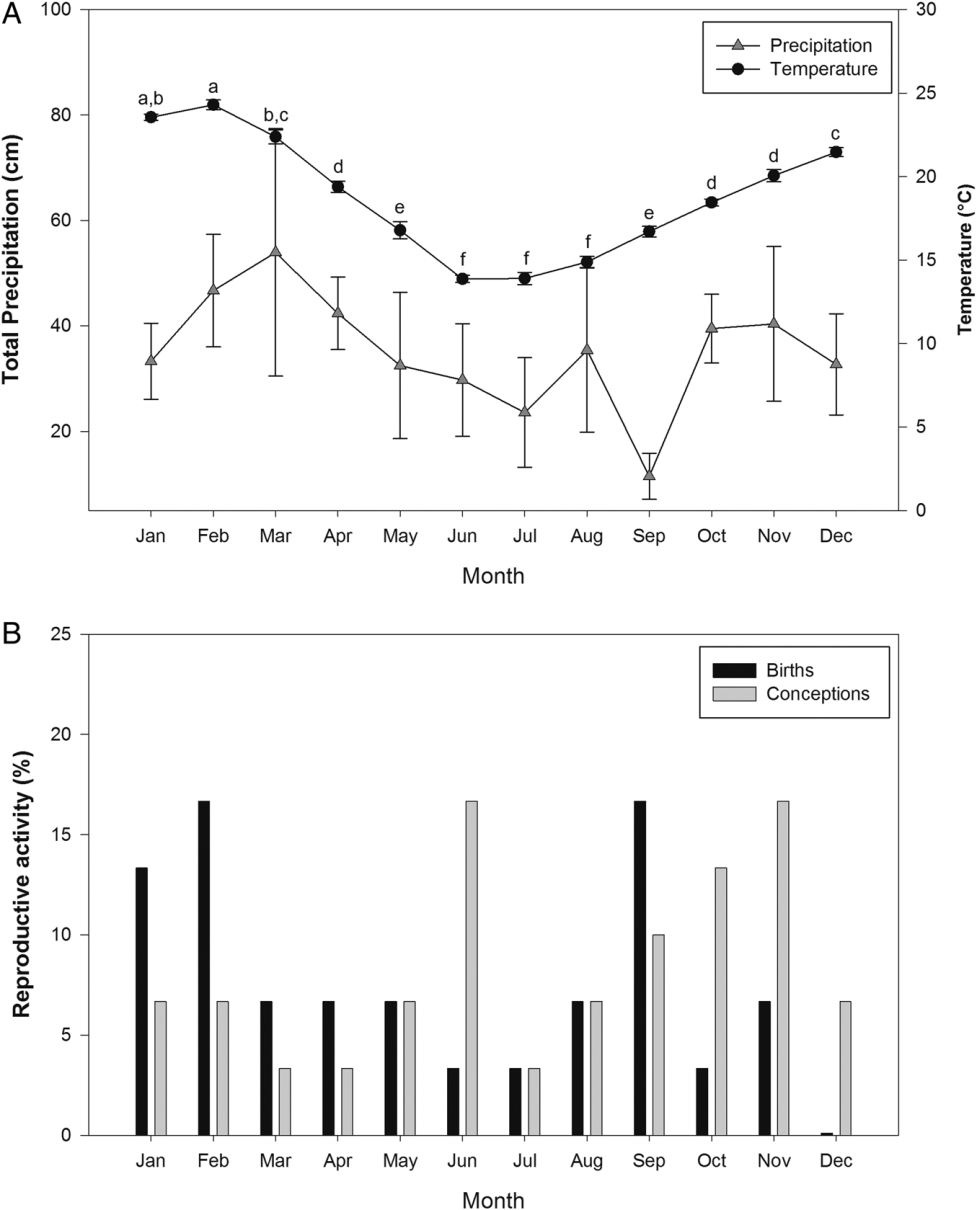
Monthly variations in total precipitation and average temperatures in Addo Elephant National Park (2005–2011; **A**) and percentage of black rhinoceros births and conceptions (**B**). Superscript letters denote significant differences (Tukey's test, *P* < 0.05).

Climate data, total monthly precipitation and average monthly rainfall were available only for 2005–2011. During that time period, total precipitation in AENP did not vary (*F*_11,64_ = 0.886, *P* < 0.56) with respect to month of the year. Over the same time period, average monthly temperature did vary (*F*_11,64_ = 133.089, *P* < 0.001) throughout the year. June, July and August had the lowest average temperatures, while January and February had the highest (Tukey's test, *P* < 0.05; Fig. 2A). Total monthly precipitation and average temperature were positively correlated (*r* = 0.594, *P* = 0.04).

### Pregnancy in Addo Elephant National Park black rhinos

Age at first parturition did not vary (*U*_15,11_ = 12.5; *P* = 0.66) between the two sections in AENP. Five Addo female black rhinos gave birth for the first time (2001–2012; *n* = 16 calves; range two to five calves per female) with an average AFP of 7.35 ± 0.71 years (range 5.8–9 years). Six Nyathi female black rhinos gave birth for the first time (1998–2011; *n* = 21 calves; range one to five calves per female) with an average AFP of 7.54 ± 0.33 years (range 7.25–9 years). Mean ICI between Addo calves was 39.10 ± 4.67 months (range 16–51 months). The rhinos in Nyathi had a shorter (*t*_8_ = −2.41, *P* = 0.04) mean ICI (27.27 ± 1.89 months; range 21–49 months) than the females in Addo.

A binomial GLM was run to determine what abiotic or biotic variables contributed to female black rhinos becoming pregnant. In the full model (Table 2), age, section, temperature and year were all significant contributors to the chance of a female black rhino being pregnant. The reduced model had a better AIC, and it predicted that the chances of collecting a faecal sample from a pregnant female were impacted by year, section and age category (Table 2). Samples were more likely to come from pregnant females in 2007 than 2008 (Tukey's test, *P* < 0.05), but there were no significant differences among any of the other years. Pregnant faecal samples were more likely to come from adult female black rhino than calves and subadults (Tukey's test, *P* < 0.05). More samples from pregnant rhinos were collected in Nyathi than from Addo. None of the other variables (season, wet/dry, average monthly temperature or total monthly precipitation) contributed to the likelihood that a faecal sample was collected from a pregnant black rhino.

**Table 2.** Binomial generalized linear model indicating which factors predicted pregnancy in female rhinoceros in Addo Elephant National Park

	Estimate ± SEM	*z* value	*P* value	AIC
Full model				
Intercept	−18.10 ± 8.85	2.04	0.04	
Age category: adult^a^				
Calf^b^	−20.74 ± 1810.00	−0.01	0.99	
Subadult^b^	−3.69 ± 1.21	−3.05	<0.01	
Precipitation	−8.16 ± 2.47	−0.33	0.74	
Season: dry				
Wet	3.26 ± 1.94	1.68	0.09	
Season: autumn				
Spring	−2.22 ± 1.77	−1.25	0.21	79.91
Summer	1.86 ± 1.47	1.26	0.21	
Winter	−4.41 ± 2.57	−1.72	0.09	
Section: Addo				
Nyathi	2.53 ± 1.10	2.30	0.02	
Temperature	−8.97 ± 0.45	−2.01	0.04	
Year: 2007^a^				
2008^b^	−5.93 ± 1.94	−3.06	<0.01	
2009^a,b^	−2.99 ± 1.47	−2.04	0.04	
2010^a,b^	−2.05 ± 1.54	−1.34	0.18	
Simplified model				
Intercept	0.98 ± 0.91	1.07	0.28	
Age category: adult^a^				
Calf^b^	−19.69 ± 1864.33	−0.01	0.99	
Subadult^b^	−3.64 ± 1.16	−3.13	<0.01	
Section: Addo				75.90
Nyathi	1.84 ± 0.83	2.22	0.03	
Year: 2007^a^				
2008^b^	−4.73 ± 1.46	−3.24	<0.01	
2009^a,b^	−2.53 ± 1.11	−2.27	0.03	
2010^a,b^	−2.06 ± 1.04	−1.99	0.05	

Abbreviation: AIC, Akaike's information criterion. Superscript letters denote significant difference (Tukey's test, *P* < 0.05).

The birth month and year was known for 30 calves (Addo, *n* = 14; and Nyathi, *n* = 16) born between January 2001 and March 2012. Most of the births (*n* = 13, 43.3%) occurred in the summer, followed by the spring (*n* = 8, 26.7%), autumn (*n* = 6, 20%) and winter (*n* = 3, 10%). By back-dating parturition dates, the month of conception was estimated. The greatest number (*n* = 13, 43.3%) of calves were conceived in the spring and the fewest (*n* = 3, 10%) were conceived in the autumn (summer, *n* = 6, 20.0%; and winter, *n* = 8, 26.7%). Most of the calves were conceived (53.3%, *n* = 16) and born (60.0%, *n* = 18) during the wet season. However, no seasonal patterns in conceptions and births were found (*r*_29_ = 0.215, *P* > 0.20) with respect to the calendar year in AENP.

Twenty-one calves were conceived and 23 born during 2005–2011, for which we have matching climate data. There was no relationship between the percentage of calves conceived each month and total precipitation (*r* = −0.128, *P* = 0.69) or average monthly temperature (*r* = −0.181, *P* = 0.57). Likewise, no relationship was found between the percentage of calves born each month and total precipitation (*r* = −0.132, *P* = 0.68). However, a positive relationship was observed between the percentage of calves born each month and mean monthly temperature (*r* = 0.609, *P* = 0.04; Fig. 2A and B).

### Female progestagen metabolite concentrations

**Figure 3.**
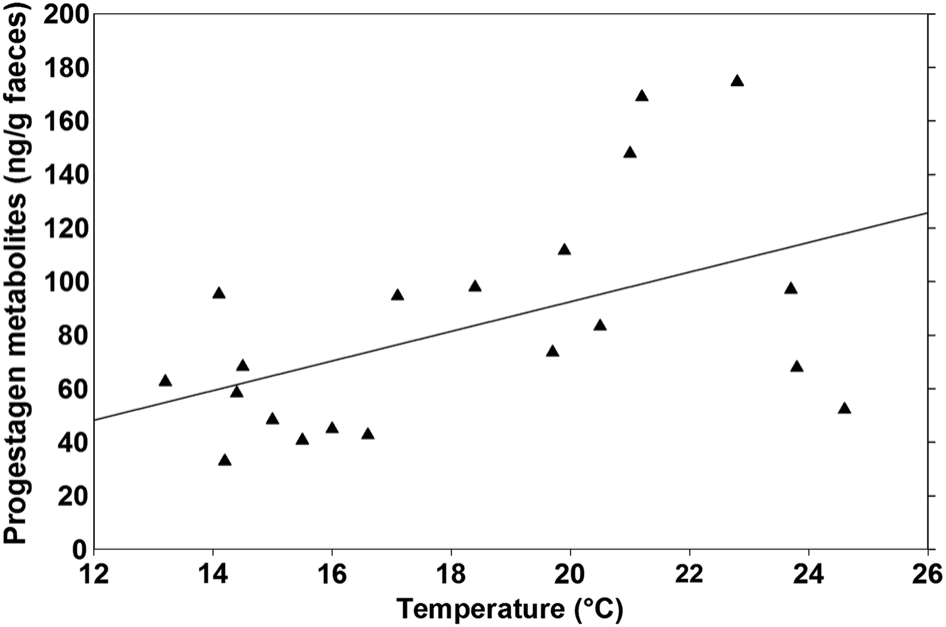
Relationship between faecal progestagen metabolite (FPM) concentrations in non-pregnant black rhinoceros and average monthly temperature in Addo Elephant National Park (FPM = −18.19 + 5.53 × temperature, *r*^2^ = 0.25, *P* = 0.03).

The FPM concentrations in non-pregnant females had a positive relationship (FPM = −18.19 + 5.53 × temperature, *r*^2^ = 0.25, *F*_1,18_ = 5.91, *P* = 0.03; Fig. 3) with average monthly temperature. In contrast, there was no relationship between FPM concentrations in non-pregnant rhinos and total precipitation (FPM = 66.85 + 0.74 × precipitation, *r*^2^ = 0.09, *F*_1,18_ = 1.78, *P* = 0.20). The FPM concentrations in pregnant rhinos were not related to the average monthly temperature (FPM = 234.72 + 21.15 × temperature, *r*^2^ = 0.03, *F*_1,16_ = 0.47, *P* = 0.50; Fig. 2) or total monthly precipitation (FPM = 586.00 + 1.68 × precipitation, *r*^2^ < 0.01, *F*_1,16_ = 0.14, *P* = 0.72).

The FPM concentrations did not vary (*H*_17_ = 26.41, *P* = 0.07) among female black rhinos within AENP. In both the full model and the reduced model, the only variable that significantly contributed to the FPM concentrations was pregnancy status (Table 3); the full model was the most parsimonious based upon AIC and Bayesian information criterion (BIC). Pregnant black rhino had higher FPM concentrations (563.82 ± 87.80 ng/g faeces, *F*_1,62_ = 55.97, *P* < 0.0001) than non-pregnant females (81.49 ± 8.23 ng/g faeces). None of the other variables (year, season, wet/dry, temperature, precipitation or age category) contributed to the FPM concentrations in AENP female black rhinos.

**Table 3.** Linear mixed-effects model predicting faecal progestagen metabolite concentrations for female black rhinos in Addo Elephant National Park, holding animal as the random effect

	Estimate ± SEM	*t* value	*P* value	AIC and BIC
Full model				
Intercept	−339.15 ± 718.05	−0.47	0.64	
Age category: adult				
Calf	−60.10 ± 128.00	−0.47	0.64	
Subadult	−9.32 ± 90.86	−0.10	0.92	
Pregnant: no				
Yes	496.81 ± 91.12	5.45	<0.01	
Precipitation	2.52 ± 2.22	1.14	0.26	
Season: dry				
Wet	15.73 ± 152.25	0.10	0.92	
Season: autumn				
Spring	228.41 ± 167.15	1.37	0.18	1025.87
Summer	49.59 ± 145.92	0.34	0.74	1061.15
Winter	223.53 ± 216.94	1.03	0.31	
Section: Addo				
Nyathi	−59.67 ± 88.06	−0.68	0.51	
Temperature	10.16 ± 35.21	0.29	0.77	
Year: 2007				
2008	78.82 ± 123.67	0.64	0.53	
2009	67.95 ± 111.45	0.61	0.54	
2010	87.60 ± 133.16	0.66	0.51	
Simplified model				
Intercept	81.49 ± 37.90	2.15	0.03	
Pregnant: no				1156.31
Yes	482.33 ± 64.46	7.48	<0.001	1177.86

Abbreviations: AIC, Akaike's information criterion; and BIC, Bayesian information criterion.

### Male androgens

Similar to the female results, the FAM concentrations did not vary (*H*_18_ = 12.14, *P* = 0.84) among male black rhinos within Addo Elephant National Park. Additionally, there was no relationship between FAM in AENP male rhinos and average monthly temperature (FAM = 95.65 + 0.25 × temperature, *r*^2^ = 0.01, *F*_1,30_ = 0.31, *P* = 0.58) or total monthly precipitation (FAM = 96.58 + 0.25 × precipitation, *r*^2^ < 0.01, *F*_1,30_ = 0.0098, *P* = 0.92).

The full and reduced model for predicting FAM concentrations from male black rhino had similar results (Table 4). However, the reduced model was the most parsimonious based upon AIC and BIC (Table 4), and it included the significant variables of year (*F*_3,118_ = 461.58, *P* < 0.001) and season (*F*_3,118_ = 2.86, *P* = 0.04), as well as age category (*F*_2,118_ = 2.69, *P* = 0.07). *Post hoc* analyses demonstrated that males had higher (Tukey's test, *P* < 0.05) FAM concentrations in the spring than in the winter; *post hoc* analyses did not demonstrate differences (Tukey's test, *P* > 0.05) in FAM concentrations among any of the other seasons (Table 4). Faecal samples collected from males in 2008 were significantly lower (Tukey's test, *P* < 0.05) than any of the three other years (Table 4). None of the other variables investigated (section of AENP, year, wet/dry season, average monthly temperature or total monthly precipitation) contributed to FAM concentrations in male black rhinos.

**Table 4.** Linear mixed-effects model predicting faecal androgen metabolite concentrations in male black rhino within Addo Elephant National Park, South Africa

	Estimate ± SEM	*t* value	*P* value	AIC and BIC
Full model				
Intercept	302.27 ± 100.15	3.02	<0.01	
Age category: adult				
Calf	−6.82 ± 15.21	−0.45	0.65	
Subadult	25.91 ± 14.09	1.84	0.07	
Precipitation	0.17 ± 0.29	0.57	0.57	
Season: dry				
Wet	23.09 ± 23.15	1.00	0.32	1523.10
Season: autumn^a,b^				1566.35
Spring^a^	5.34 ± 22.56	0.24	0.81	
Summer^a,b^	4.28 ± 16.81	0.25	0.80	
Winter^b^	−51.16 ± 26.37	−1.94	0.05	
Section: Addo				
Nyathi	−10.73 ± 14.59	−0.74	0.47	
Temperature	−7.74 ± 5.13	−1.51	0.13	
Year: 2007^a^				
2008^b^	−89.92 ± 22.65	−3.97	<0.01	
2009^a^	−42.95 ± 21.66	−1.98	0.05	
2010^a^	−29.47 ± 25.06	−1.18	0.24	
Simplified model				
Intercept	148.09 ± 24.08	6.15	<0.001	
Age category: adult				
Calf	−8.04 ± 14.30	−0.56	0.57	
Subadult	26.84 ± 13.18	2.04	0.04	
Season: autumn^a,b^				
Spring^a^	26.93 ± 17.30	1.56	0.12	1536.90
Summer^a,b^	0.76 ±13.50	0.06	0.96	1568.94
Winter^b^	18.38 ± 15.45	−1.19	0.24	
Year: 2007^a^				
2008^b^	−86.06 ± 22.28	−3.86	<0.01	
2009^a^	−41.85 ± 21.09	−1.98	0.05	
2010^a^	−32.61 ± 23.92	−1.36	0.18	

Abbreviations: AIC, Akaike's information criterion; and BIC, Bayesian information criterion. Superscript letters denote significant difference (Tukey's test, *P* < 0.05).

## Discussion

Although we predicted that three factors, i.e. variations in the number of elephants, predators and tourists, would affect reproductive success of black rhino, few differences were discovered between the AENP Addo and Nyathi subpopulations. No variations between the subpopulations were found with respect to AFP, FPM and FAM concentrations. The only differences between rhinos in the two sections were that females in Addo had a longer inter-calving interval, and we had a lower likelihood of collecting samples from pregnant rhinos in Addo. The present study did not directly test the influence of differences between the two sections of AENP and thus could not determine for certain if the impacts of competitors, predators and/or tourists contributed to the differences in ICI and chances of pregnancy between Nyathi and Addo.

We were unable to point to the exact cause of variability in ICI and pregnancy rates between the two subpopulations of black rhino in AENP. We had greater sampling success from rhinos in Nyathi than Addo, which may have contributed to differences in the chances of finding pregnancy samples between the two sections. However, we followed the same number of pregnant females in both sections (*n* = 4) and only one more pregnancy in Nyathi (*n* = 5) because one female was pregnant with two different calves during the course of our study. Thus, we do not believe that sampling intensity is the sole reason for the differences found between sections, because additional data comparing the subpopulations suggest that the health of the animals in Addo may be compromised. For instance, black rhinos in Addo had fewer ciliate species present in their faeces compared with Nyathi rhinos; these beneficial organisms aid in digestion and serve as a proxy for overall nutritional health (J. Aronoff and T. Gillespie, unpublished data). Additionally, a physiological response to environmental stressors in AENP was documented, with Addo black rhinos having higher concentrations of faecal glucocorticoid metabolite concentrations than individuals in Nyathi (Santymire *et al.*, 2011). The time intervals at which Addo rhinos slept (20.00–24 00 h) were shorter than those in Nyathi (20.00–04.00 h; Santymire *et al.*, 2012). Likewise, AENP rhino shifted their activity patterns in response to elephant presence (C. Tambling, unpublished data) and diet in areas where elephants have altered the vegetation (Landman *et al.*, 2013; Landman and Kerley, in press). Thus, biotic interactions are influencing the behaviours of black rhinos within the subpopulations. Combined, these patterns relative to endocrinology, sleep and parasitic infection rates create an overall assessment of the AENP black rhino subpopulations and suggest that biotic and anthropogenic disturbances in Addo may be negatively impacting black rhino health.

Furthermore, the environmental pressures could be negatively impacting the physiology of Addo black rhinos by contributing to longer ICIs through higher rates of aborted fetuses and/or neonatal predation prior to discovery of the calf (Hitchins and Anderson, 1983). Abortions of fetuses are not uncommon; they have been documented in at least two other free-ranging populations of black rhinoceros (Garnier *et al.*, 2002; MacDonald *et al.*, 2008). Pregnancy in black rhinos cannot be diagnosed until the significant rise in FPM after 3 months of gestation (Schwarzenberger *et al.*, 1993, 1996; Berkeley *et al.*, 1997; Garnier *et al.*, 1998; MacDonald *et al.*, 2008), and these early stages are a vulnerable period for black rhino pregnancies (Garnier *et al.*, 2002). Thus, female black rhino may be aborting prior to our ability to detect the pregnancy via faecal endocrine monitoring. We now know that it is possible to monitor non-invasively the oestrous cycle activity, including pregnancies, of black rhinos in AENP based upon the location and length of their scraped faeces (Freeman *et al.*, 2014). Insight into these behaviours may help population managers to detect lost pregnancies more easily. Additionally, black rhino calves <3 months of age are vulnerable to hyena predation, which can contribute to ICIs >40 months (Hitchins and Anderson, 1983). Two calf mortalities were recorded in the reintroduced population of *D. b. michaeli* in AENP (Hall-Martin and Penzhorn, 1977); one was trampled by a bull buffalo at 7 months of age, and another was attacked by a pack of dogs (*Canis familiaris*) at 3 months of age. Unlike Nyathi, the Addo section has resident hyena and lions that could be preying upon neonatal black rhinos before they are seen. Greater competition for resources with elephants, disturbance by tourists and predation in Addo could be causing reproductive stress and contributing to longer ICIs.

The ICI and AFP found within the two AENP black rhino subpopulations were similar to those found in other studies of free-ranging black rhino. The population of *D. b. michaeli* reintroduced into AENP had a mean ICI of 35 months (range 24–52 months; Hall-Martin and Penzhorn, 1977). The shortest interval on record was 24 months, with conception taking place 2 months after a calf was killed. The age at which two of the original AENP rhinos produced their first calves was 8.5 and 8.0 years, respectively; females were first observed mating at 4.5 years of age (Hall-Martin and Penzhorn, 1977). In Hluhluwe Umfolozi, ICI ranged from 20 to 80 months (Hluhluwe, mean 44.5 months; and Corridor/Umfolozi, mean 30.6 months), and two females aged 6.0 and 8.5 years, respectively, produced their first calves (Hitchins and Anderson, 1983).

The shortest ICI recorded in the present study for the AENP subpopuations was 16 months. With a documented gestation length of 450–456 days in free-ranging rhino (Garnier *et al.*, 1998) and 454–470 days in zoo-managed females (Schwarzenberger *et al.*, 1993), this means that the AENP females became pregnant within a month of giving birth. Follicular maturation resumes 2–4 weeks after parturition in some zoo (Schwarzenberger *et al.*, 1993; Berkeley *et al.*, 1997) and free-ranging female black rhino (Garnier *et al.*, 2002), which could enable those individuals to become pregnant. However, it has been suggested that most wild females have two to eight oestrous cycles prior to conception of the next calf, leading to an ICI of 19–30 months (Garnier *et al.*, 2002).

The AENP female black rhinos were pregnant all times of the year. Although there was no relationship between conceptions or births and total monthly precipitation in AENP, non-pregnant females had higher FPM concentrations and a larger percentage of calves were born in the months with higher average temperatures. These trends suggest that warmer months are a favourable time of year in AENP for black rhinos to conceive and have calves. Faecal progestagen concentrations would be highest in non-pregnant females going through oestrus. Additionally, females would be giving birth when vegetative quality can support the demands of lactation. No differences were found in total monthly precipitation in AENP over the course of this study. Water is pumped to supply man-made waterholes, providing animals in AENP with fresh water throughout the year. Additionally, AENP herbivores acquire moisture by eating the succulent spekboon (*Portulacaria afra*) thicket (Penzhorn *et al.*, 1974). Thus, it is not surprising that the precipitation had little impact on reproductive rates in the AENP black rhino. Likewise, faecal progestagen metabolite concentrations in African elephants from AENP had no relationship with average monthly precipitation (Freeman *et al.*, 2013). However, there were seasonal variations in temperature, with June–August being the coolest months and January–February the hottest. Seasonal patterns in births among the AENP *D. b. bicornis* population were similar to those observed in the earlier *D. b. michaeli* population. The majority (66%) of *D. b. michaeli* births occurred in the summer months (Hall-Martin and Penzhorn, 1977), which also had the greatest percentage of *D. b. bicornis* births in the present study. Yet, conception patterns between the two studies differed. Hall-Martin and Penzhorn (1977) reported that no black rhino mated in AENP during May–July. In contrast, back-dating parturition dates from the present study predicted that eight *D. b. bicornis* calves were conceived in those same months; however, only one calf was conceived during March–April.

Similar to AENP, matings in Hluhluwe/Umfolozi occur throughout the year, yet 65% took place between October and December (Hitchins and Anderson, 1983). Conceptions in Hluhluwe/Umfolozi showed a bimodal distribution, with peaks in October–November and April–July, possibly cued by light stimulus (Hitchins and Anderson, 1983). Extended inter-oestrous intervals (April–June) in nulliparous females coincide with the autumnal equinox in the southern hemisphere and decreasing day length in Zimbabwe (Garnier *et al.*, 2002). Additionally, precipitation is related to reproduction in Zimbabwe, with 62% of births occurring in the later portion of the rainy season (February–May), and conceptions (November–February) coinciding with the early part of the rainy season (Garnier *et al.*, 2002). Timing of gestations at the end of the rainy season in Zimbabwe ensures that temperature and food resources are optimum for the growth and survival of young (Garnier *et al.*, 2002). Although black rhino conceptions may be cued by light, temperature or precipitation, the prolonged gestation (455 days) and calving intervals are probably too infrequent to enable the species to evolve an optimal breeding season (Hitchins and Anderson, 1983). Additionally, environmental conditions at birth may not be critical enough to drive evolution of reproductive seasonality, particularly when lactation persists over multiple seasons (Hitchins and Anderson, 1983).

Only a few factors were related to faecal endocrine metabolite concentrations in AENP black rhino. Pregnancy status was the only variable that contributed to FPM concentrations in female black rhino. After the first 3 months of gestation, FPM concentrations rise significantly and are 4–10 times higher than non-pregnant values (Schwarzenberger *et al.*, 1993, 1996; Berkeley *et al.*, 1997; Garnier *et al.*, 1998; MacDonald *et al.*, 2008). This rise is so dramatic that it allows pregnancy to be diagnosed from a single sample and to be determined visually without the need for expensive equipment (MacDonald *et al.*, 2008). The 7-fold difference between FPM concentrations from pregnant and non-pregnant samples in the present study may have masked any other factors that contributed to FPM concentrations.

Male black rhinos in AENP had the highest FAM concentrations in spring. Thus, the peak FAM production preceded the peak in conceptions, 27% (spring) and 43% (summer), in comparison to 20% in autumn and 10% in winter. Likewise, higher FAM concentrations in male white rhino (*Ceratotherium simum simum*) managed at a South African game farm also coincide with a greater frequency of conceptions during the rainy season (Kretzschmar *et al.*, 2004). Although these results suggest that higher FAM concentrations may facilitate sperm production in African rhinos, the minimal hormone concentrations to promote libido and spermatogenesis have not been established in these species (Christensen *et al.*, 2009). Kretzschmar *et al.* (2004) suggested that presence of female white rhino prompted the higher FAM concentrations and inter-sexual aggression associated with mating behaviour. In zoo populations, serum testosterone concentrations in male black and white rhino are positively correlated with the number of female conspecifics, and testosterone concentrations in black rhino are also positively correlated with the number of males (Christensen *et al.*, 2009). These results help to support the idea that black rhino are more social than previously documented and that their solitary nature may be an artifact of the present small population size (Garnier *et al.*, 2001; Christensen *et al.*, 2009).

### Conclusions

Recent findings about sociality in black rhino and factors that impact reproduction could help set management parameters that promote population growth. Ensuring that all free-ranging black rhinos of reproductive age are reproductively successful is critically important as poaching rates continue to rise (Emslie, 2012) in native habitat, such as South Africa. Managers of black rhino populations must consider current needs as well as the impact of management decisions on the future success of populations (Hitchins and Anderson, 1983). Results from the present study suggest that environmental pressures from competitors, predators and/or tourists may negatively impact the health and reproductive success of black rhino populations. Future studies of other populations where it is possible to separate the impact of these environmental stressors should be conducted. Traditional techniques for monitoring free-ranging populations include gathering crucial information for assessing population demographics, such as age at first parturition and inter-calving intervals. We suggest that future research should also gather physiological data (Cooke *et al.*, 2013) by employing non-invasive faecal endocrine methods, which enhance these traditional methods through more accurate assessment of fertility rates in a population. Additionally, faecal endocrine monitoring can help to detect how environmental conditions, such as predators or competitors, reduce reproductive success through changes in hormone values and increases in fetal loss or perinatal mortality (Garnier *et al.*, 1998). This knowledge can facilitate early detection of any changes in the population demographics that will need to be addressed through management decisions (Cooke *et al.*, 2013), such as translocations and introductions of rhinos as well as possible competitors and predators. Owing to the high costs and logistical difficulties associated with black rhino management (Garnier *et al.*, 1998), knowledge gained about the reproductive status of populations and a better understanding of factors that affect reproductive health can optimize management efficiency.
